# Minimally invasive treatment strategy for severe congenital pulmonary valve insufficiency and pulmonary artery aneurysmal dilatation: a case report

**DOI:** 10.3389/fcvm.2026.1778125

**Published:** 2026-04-22

**Authors:** Daokuo Zheng, Jingjing Chen, Yanfei He, Bangtian Peng, Zhenwei Ge

**Affiliations:** 1 Department of Cardiovascular Surgery, Henan Chest Hospital, Zhengzhou University, Zhengzhou, China; 2 Department of Cardiovascular Surgery, Henan Provincial People's Hospital, Fuwai Central China Cardiovascular Hospital, Zhengzhou University, Zhengzhou, China; 3 Department of Medical Imaging, Henan Chest Hospital, Zhengzhou University, Zhengzhou, China

**Keywords:** congenital valvular disease, minimally invasive treatment strategy, myxomatous degeneration, pulmonary artery aneurysmal dilatation, pulmonary valve insufficiency

## Abstract

Pulmonary valve insufficiency most commonly occurs following various precordial interventions, including repair of tetralogy of Fallot, balloon dilatation for pulmonary stenosis, and surgical or percutaneous treatment for pulmonary atresia with intact ventricular septum. Isolated congenital pulmonary valve insufficiency is rare, and insufficiency secondary to myxomatous degeneration of the pulmonary valve is even rarer. We report the case of a 16-year-old female with severe pulmonary valve insufficiency and aneurysmal dilatation of the pulmonary artery caused by myxomatous degeneration of the pulmonary valve. The patient underwent minimally invasive surgical treatment, and no regurgitation was observed at one-year follow-up.

## Case report

A 16-year-old female was admitted with a chief complaint of a heart murmur first detected three months prior during a routine physical examination. She was asymptomatic. Her blood pressure was 110/73 mmHg, heart rate 71 bpm, and transcutaneous oxygen saturation 98%. A grade 3/6 diastolic murmur was audible in the second intercostal space at the left sternal border.

**Figure 1:**
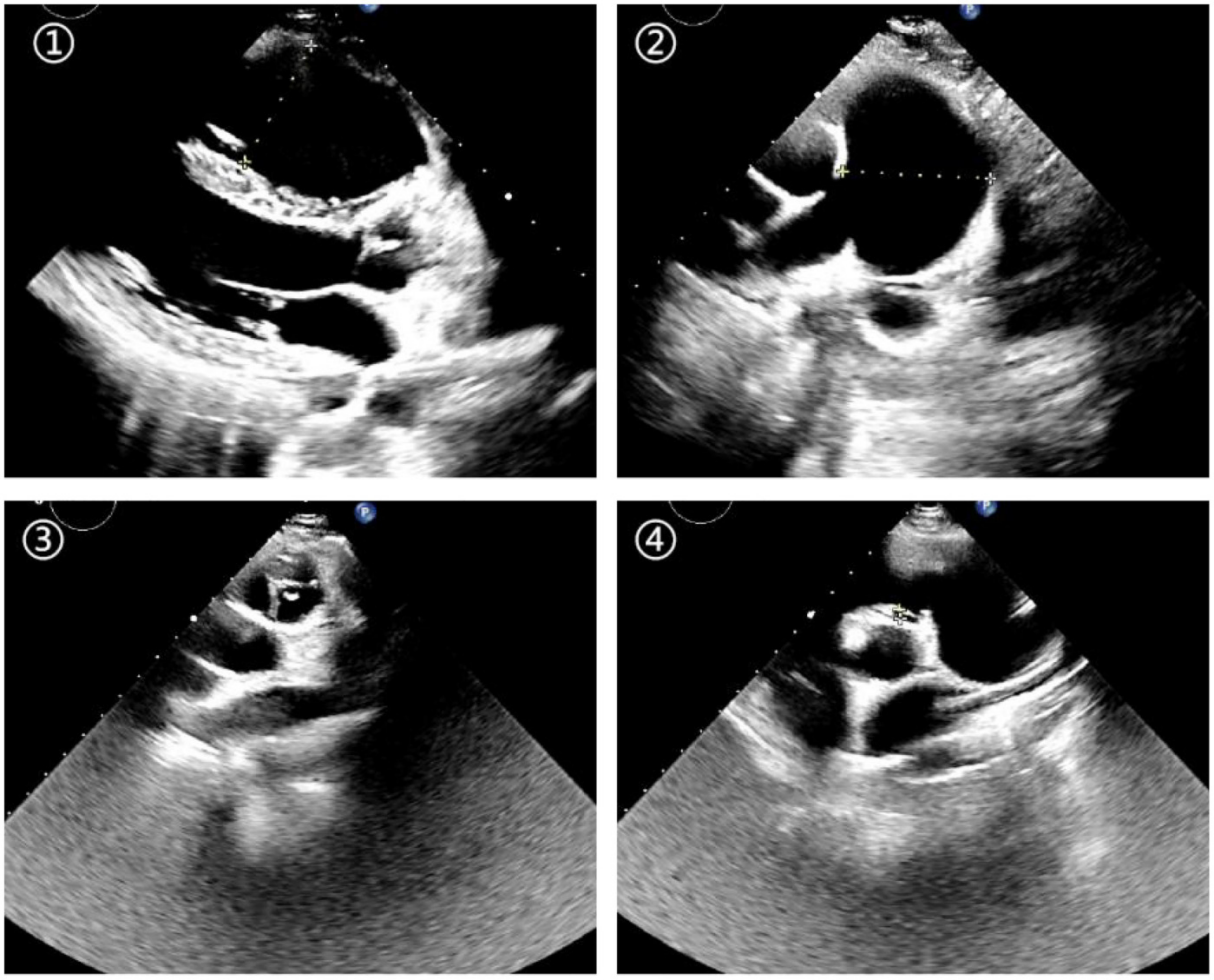
Echocardiography showing right ventricular dilatation (①), pulmonary artery dilatation (②), anterior and right pulmonary valve leaflets (③), and downward displacement of the left pulmonary valve leaflet (④).

**Figure 2:**
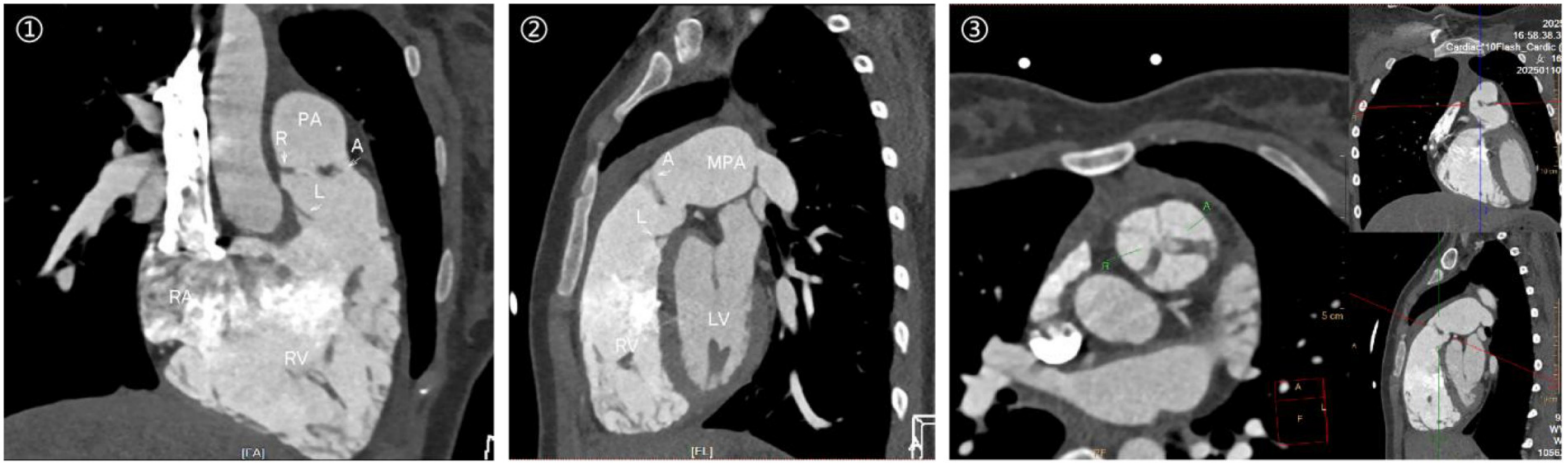
Cardiac computed tomography angiography demonstrating aneurysmal dilatation of the main pulmonary artery (diameter 44 mm, arrow) and marked right ventricular enlargement.

Echocardiography revealed a three-leaflet pulmonary valve with redundant, thickened leaflets and echogenic edges. The valve opened adequately but failed to coapt properly. The regurgitation jet area measured 12.6 cm^2^, and the coaptation defect measured 12 mm, accounting for more than 60% of the width of the right ventricular outflow tract. The pulmonary valve annulus measured 31 mm in diameter, and the main pulmonary artery was aneurysmal, with an internal diameter of 44 mm. The left and right pulmonary artery branches measured 14 mm and 15 mm, respectively (Figure 1). Electrocardiography showed sinus arrhythmia and incomplete right bundle branch block. Chest radiography revealed clear lung fields and an enlarged right heart. Cardiac computed tomography angiography confirmed severe pulmonary valve insufficiency, aneurysmal dilatation of the pulmonary artery, and right ventricular enlargement (Figure 2).

Following comprehensive evaluation, the patient underwent pulmonary valve and pulmonary artery replacement under general anesthesia and cardiopulmonary bypass. The neovalve leaflets were constructed from a 0.1-mm expanded polytetrafluoroethylene (ePTFE) membrane using a size 23 Ozaki template and were implanted into a size 22 ePTFE conduit (Gore-Tex; W. L. Gore & Associates, Flagstaff, AZ, USA) to create a valved conduit composite graft.

## Fabrication of the pulmonary valved conduit

The valved conduit was assembled on the back table after systemic heparinization. The diameter of the ePTFE conduit was selected based on the patient's body surface area and the preoperative CT-measured diameter at the pulmonary artery bifurcation. The selected conduit was turned inside out.

The dimensions of the artificial leaflets were calculated as follows:

Leafletwidth=(Conduitdiameter×π)/3

Leafletheight=Width×0.75

An Ozaki template matching the calculated width was selected. Three identical leaflets were cut from a 0.1-mm ePTFE membrane using the chosen template and were sutured with 6-0 polypropylene (Prolene) sutures according to the following steps:

### Nine-point positioning

The leaflets were initially secured at three equidistant nadir points (points 1, 2, 3). Suturing then proceeded symmetrically toward the commissures (points 4, 5, 6) and finally to the three commissural apex points (points 7, 8, 9).

### Suturing the leaflet to the conduit

Beginning at the leaflet nadir, a running suture was used to attach the leaflet to the conduit wall. A 2:1 suture ratio was applied near the nadir to create excess tissue for coaptation, transitioning to a 1:1 ratio toward the commissures to maintain the appropriate annular curvature. The nadir suture was tied and secured.

### Commissural fixation

When the suture reached the commissural area (approximately 5 mm in height), a mattress suture was placed to anchor the commissure, followed by continuous running sutures. The suture ends were passed through the conduit wall and tied externally. Adequate commissural height was maintained to reduce diastolic leaflet stress and minimize regurgitation.

### Suturing adjacent leaflets at the commissure

After completing the continuous suture to the commissure, the two adjacent leaflets were joined with a mattress suture.

### Commissural reinforcement

A second running suture was used to secure the two leaflets together along the commissural line down to the annular level. Care was taken to avoid incorporating residual native leaflet tissue to prevent late leaflet dehiscence.

### Final commissural anchoring

**Figure 3:**
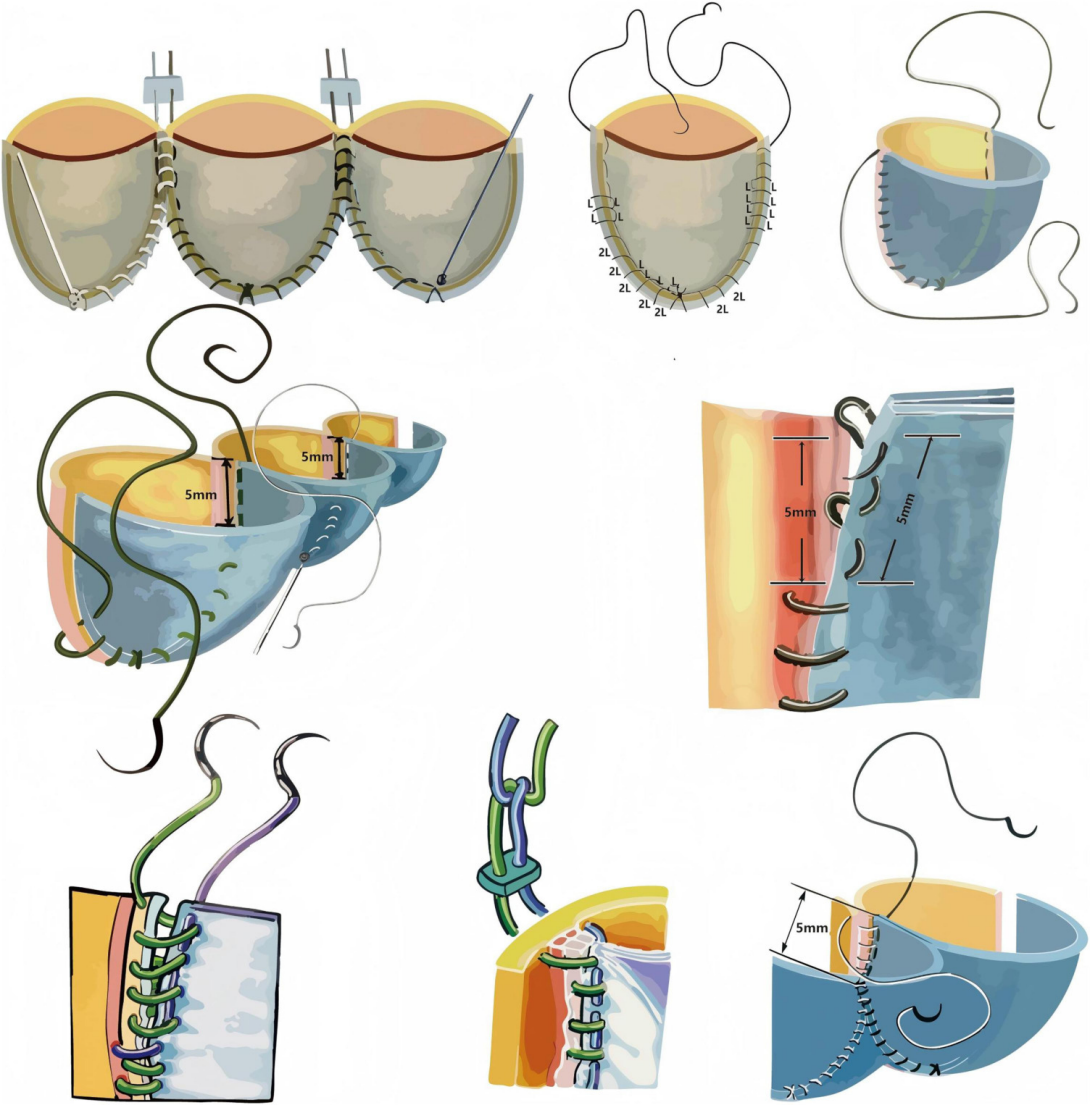
Step-by-step fabrication of the trileaflet ePTFE valved conduit.

**Figure 4:**
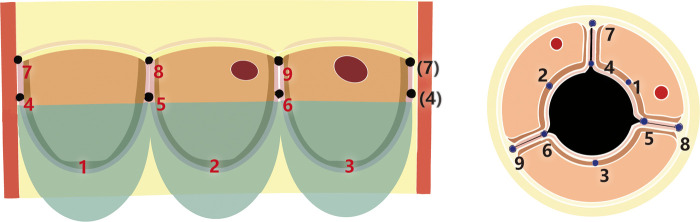
Final appearance of the hand-sewn trileaflet ePTFE valved conduit and verification of leaflet coaptation prior to implantation.

Both commissural sutures were passed through the conduit wall and tied externally. The valved conduit was then turned right-side out, and leaflet coaptation was visually verified before final implantation. The detailed fabrication steps for the Fabrication of the pulmonary valved conduit are shown in Figures 3, 4.

## Surgical technique

The patient was placed in the left lateral decubitus position. A right anterolateral thoracotomy was performed through the fourth intercostal space. The pericardium was incised and suspended.

Cardiopulmonary bypass was established through the same incision. Arterial cannulation was performed on the ascending aorta, and the superior vena cava was cannulated directly. For inferior vena cava drainage, a cannula was inserted through a separate incision below the thoracotomy. A split sternal retractor was used to optimize exposure without conversion to full sternotomy.

After aortic cross-clamping, Del Nido cardioplegia was administered antegradely via the aortic root. The fossa ovalis was incised to place a left ventricular vent.

The main pulmonary artery was opened longitudinally down to the annulus. The native pulmonary valve leaflets (anterior, right, and left—the latter extending onto the interventricular septum) were completely excised.

**Figure 5:**
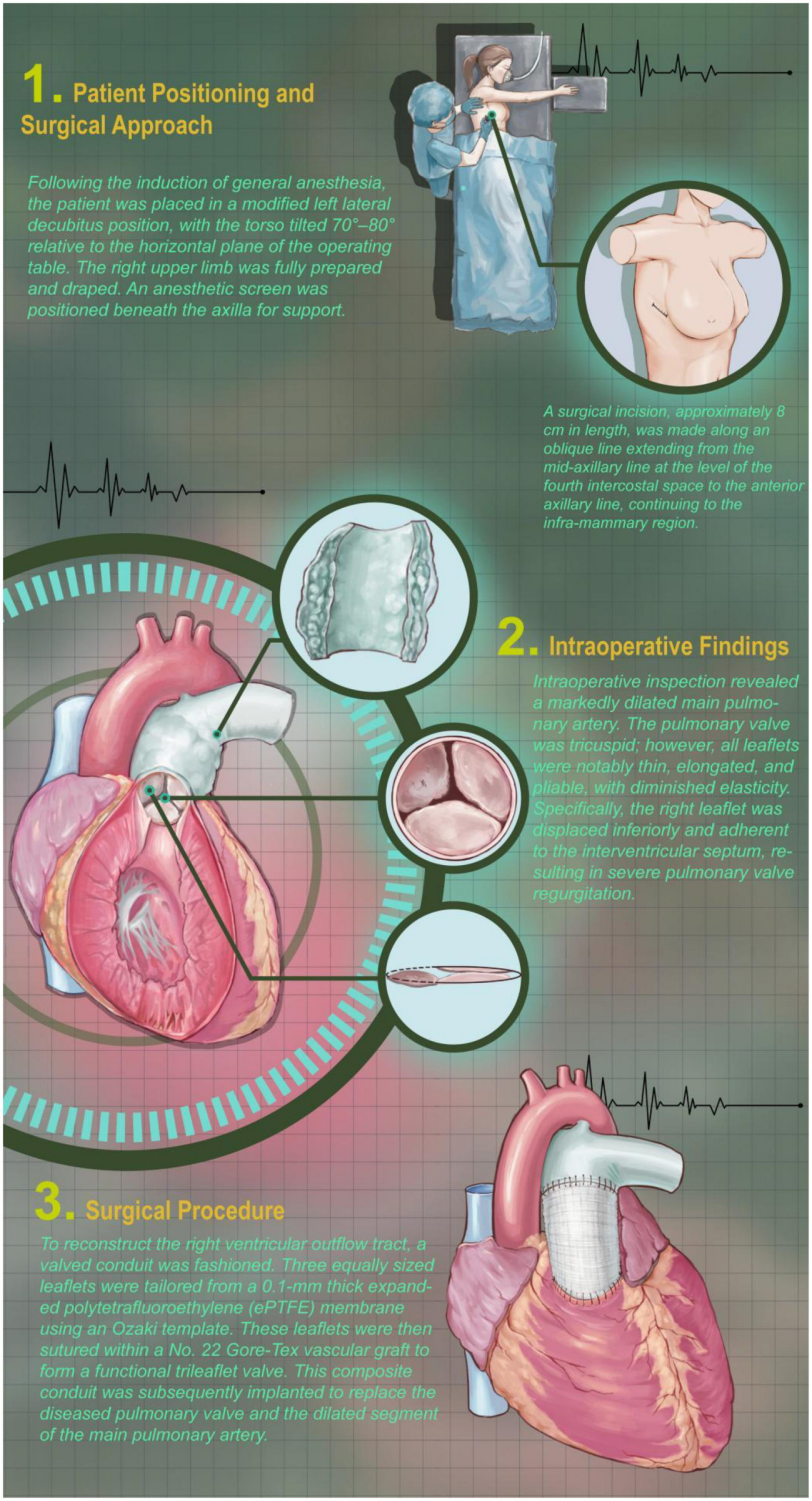
Intraoperative photographs illustrating the right anterolateral thoracotomy incision, exposure of the right ventricular outflow tract, and completed anastomosis of the ePTFE valved conduit.Please let me know if any further adjustments are needed.

The distal end of the hand-sewn valved conduit was anastomosed to the distal main pulmonary artery using a running 5-0 Vicryl suture. The proximal main pulmonary artery was trimmed, and the proximal end of the conduit was anastomosed to the right ventricular outflow tract with a running 5-0 Vicryl suture. The key surgical steps and procedures for the right ventricular outflow tract reconstruction performed in this case are illustrated in Figure 5.

During rewarming, thorough de-airing was performed via the left ventricular vent and the ascending aorta. The heart resumed spontaneous sinus rhythm at 80 bpm after cross-clamp removal. The atrial septal incision and right atriotomy were closed with running 5-0 polypropylene sutures.

Intraoperative transesophageal echocardiography confirmed satisfactory function of the prosthetic pulmonary valve with no regurgitation.

## Intraoperative findings and postoperative course

Intraoperative inspection revealed that the right and anterior pulmonary valve leaflets were in normal position, while the left leaflet was displaced downward and attached to the interventricular septum. A 3-mm perforation was noted in the mid-portion of the left leaflet. All three leaflets were elongated, soft, and functionally insufficient. The pulmonary valve annulus was dilated, and the main pulmonary artery was aneurysmal.

**Figure 6:**
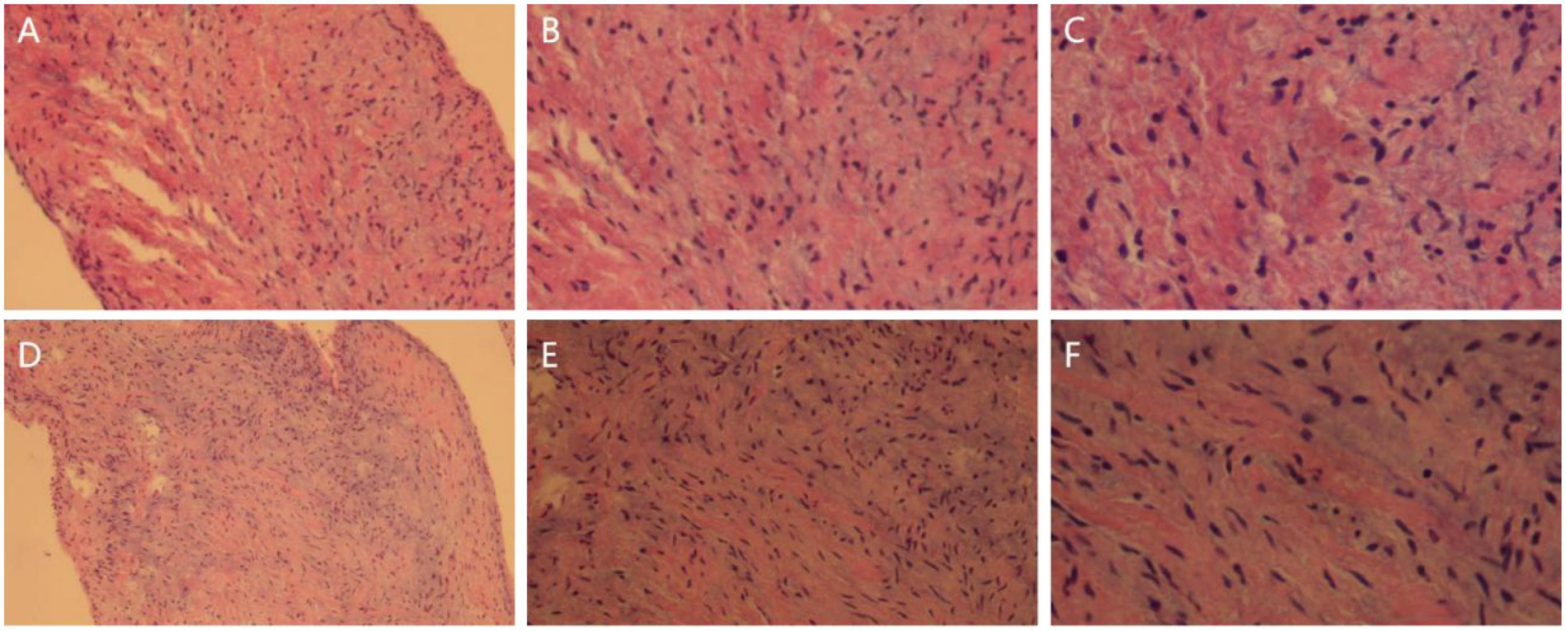
Hematoxylin-eosin staining of the excised pulmonary valve leaflet showing myxomatous degeneration within the tissue interstitium. **(A,D)** Original magnification  × 100; **(B,E)** × 200; **(C,F)** × 400.

Postoperative pathological examination confirmed myxomatous degeneration of the pulmonary valve (Figure 6).

The patient was extubated 12 h postoperatively and transferred from the intensive care unit to the general ward on postoperative day 2. She was discharged one week after surgery.

Postoperative echocardiography showed good pulmonary valve function with no regurgitation. At one-year follow-up, the valve continued to function well without evidence of regurgitation or stenosis. Long-term follow-up is planned to provide further insights for future clinical practice.

## Comment

Pulmonary valve insufficiency most commonly occurs after surgical or interventional procedures for congenital heart disease, including repair of tetralogy of Fallot (1), balloon dilatation for isolated pulmonary stenosis (2), and surgical treatment of pulmonary atresia with intact ventricular septum (3). Rare cases secondary to thoracic trauma or congenital absence of a single pulmonary valve leaflet have also been reported (4, 5).

Myxomatous degeneration of the cardiac valves is a non-inflammatory, non-atherosclerotic degenerative process characterized by pathological accumulation of mucopolysaccharides within the valvular interstitial matrix (6, 7). This process leads to fragmentation of elastic fibers and collagen bundles, ultimately resulting in valvular dysfunction. Although it most commonly affects left-sided heart valves, myxomatous degeneration involving the pulmonary valve in the low-pressure right heart environment is exceedingly rare (8). Consequently, there is a lack of consensus regarding its natural history, rate of progression, and optimal timing for surgical intervention.

To the best of our knowledge, congenital pulmonary valve insufficiency with aneurysmal dilatation of the pulmonary artery secondary to primary myxomatous degeneration involving all three leaflets—as presented in this case—has not been previously documented.

Mild to moderate pulmonary valve insufficiency is generally well tolerated due to compensatory right heart mechanisms and may remain asymptomatic for many years (9). However, as regurgitation severity progresses, chronic volume overload leads to right ventricular dilatation and dysfunction, exercise intolerance, secondary tricuspid regurgitation, ventricular arrhythmias, and even sudden death (10). These life-threatening consequences underscore the importance of timely surgical intervention in patients with severe pulmonary valve insufficiency.

Pulmonary valve replacement is a well-established intervention for pulmonary valve disease and is particularly common in the post-cardiotomy setting (11, 12). Nevertheless, surgical management of congenital pulmonary valve insufficiency remains heterogeneous and is largely individualized. Inoue et al. (5) reported single-leaflet replacement in a patient with congenital absence of one leaflet and two morphologically normal leaflets. Vaidyanathan et al. (13) performed pulmonary valve replacement in a 4-year-old child with Uhl's anomaly-associated pulmonary valvular agenesis syndrome. Parale et al. (14) described a young female with posterior pulmonary valve agenesis, dysplastic residual leaflets, and aneurysmal pulmonary artery dilatation who underwent valve replacement using a bioprosthetic conduit.

While homografts and bovine jugular vein conduits remain commonly used options for right ventricular outflow tract reconstruction, their applicability in our setting is substantially limited. Homografts are constrained by donor availability, whereas bovine jugular vein conduits carry inherent risks of late calcification, infection, and consequent reoperation. Thus, a more durable alternative is needed. In this context, expanded polytetrafluoroethylene has emerged as a promising material owing to its excellent biocompatibility, resistance to calcification, and long-term durability. Since its initial description by Yamagishi et al. (15–17), the surgical technique for ePTFE-based valved conduit construction has continued to evolve.

In contrast to previously reported cases and conventional conduit options, the present case exhibits several distinctive features that necessitated a different reconstructive strategy.

### First, regarding etiology

Unlike congenital agenesis or hypoplasia, our patient presented with primary myxomatous degeneration involving all three pulmonary valve leaflets. This inherent abnormality of the connective tissue matrix renders any valve-sparing or partial resection approach unreliable, given the progressive nature of the underlying pathology. This pathology fundamentally justifies the use of a non-biological, degradation-resistant material such as ePTFE.

### Second, regarding anatomical involvement

While prior cases predominantly involved isolated leaflet absence or preserved remnant leaflet tissue, our patient demonstrated global, trileaflet involvement with severe regurgitation, accompanied by significant pulmonary root dilatation and main pulmonary artery aneurysm. This combination of diffuse valvular destruction and annular–arterial enlargement not only precludes the use of commercially available fixed-size conduits but also fundamentally excludes the anatomic feasibility of transcatheter pulmonary valve implantation (TPVI). The substantially dilated and thin-walled native annulus and main pulmonary artery lack a sufficiently healthy landing zone, rendering TPVI at high risk for valve migration and paravalvular leak. Concurrent ascending aortic dilatation further increases the risk of vascular injury during interventional manipulation.

### Third, regarding material and technical considerations

Unlike the bioprosthetic or homograft conduits used in prior reports—each associated with well-documented limitations in availability, durability, or structural deterioration in young patients—our strategy employed a hand-sewn trileaflet ePTFE valved conduit. This approach offers three distinct advantages tailored to the present case:

1. Lifelong durability — ePTFE exhibits excellent resistance to calcification and biodegradation, providing the young patient with the best available protection against reoperation;
2. Individualized customization — conduit diameter, length, and geometry can be precisely tailored intraoperatively to match the dilated pulmonary root dimensions;
3. Concomitant management capability — through a minimally invasive incision, concurrent pulmonary arterioplasty can be performed to address the dilated main pulmonary artery.

### Fourth, the deliberate exclusion of transcatheter options

During the decision-making process, we systematically evaluated the applicability of TPVI. Beyond the aforementioned anatomical limitations, TPVI is inherently a “valve-in-valve” implantation that does not remove the diseased native leaflets or subvalvular structures, nor does it correct the geometric abnormalities resulting from root dilatation. In a 16-year-old patient, such a palliative intervention not only leaves a clear anatomic substrate for long-term complications but also carries the inevitable risk of structural bioprosthetic deterioration. Should future valve failure occur, subsequent management—particularly conversion to surgical replacement—would be rendered extremely complex by the presence of a valve-in-valve composite and potential calcification of the right ventricular outflow tract, substantially increasing the risk and difficulty of reoperation.

Therefore, we opted for a single-stage, anatomically curative surgical reconstruction using a hand-sewn trileaflet ePTFE valved conduit, aiming to provide the patient with the most durable and stable long-term solution. The present case demonstrates that, in the setting of trileaflet degeneration with concomitant root dilatation, this approach represents a physiologically sound, anatomically adaptable, and durable alternative. This report adds to the growing body of evidence supporting the expanded application of ePTFE reconstruction in complex right-sided valvular pathology.

## Limitations

**Figure 7:**
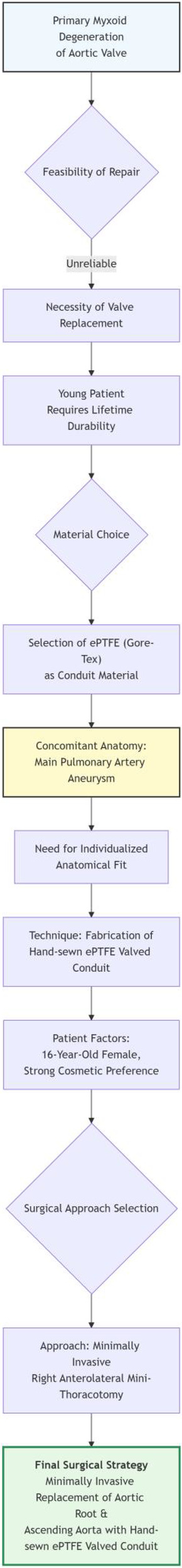
Surgical Decision Flowchart. This flowchart illustrates the clinical decision-making process for primary myxomatous degeneration of the pulmonary valve in young patients, including evaluation of repair feasibility, determination of replacement necessity, material selection (ePTFE), consideration of main pulmonary aneurysm-like dilation, personalized fit requirements, fabrication of manually sutured grafts, selection of surgical approach, and ultimately the implementation of minimally invasive valve replacement and main pulmonary artery replacement.

Owing to the surgical team's intense focus on performing complex and delicate maneuvers within a confined minimally invasive field, continuous intraoperative imaging documentation could not be obtained. This represents a limitation of the present study. To mitigate this shortcoming, we have provided an exhaustive step-by-step description of the surgical technique and supplemented it with professionally rendered schematic illustrations, thereby ensuring that the key anatomical details and technical principles are conveyed with sufficient clarity and reproducibility. The step-by-step treatment decision-making algorithm for selecting the surgical approach is illustrated in Figure 7.

## Data Availability

The datasets presented in this study can be found in online repositories. The names of the repository/repositories and accession number(s) can be found in the article/Supplementary Material.
